# Supplementary material for: Real-world evidence with a retrospective cohort of 15,968 COVID-19 hospitalized patients suggests 21 new effective treatments
Source: Virol J. 2023 Oct 6;20:226. doi: 10.1186/s12985-023-02195-9 (PMC10559601; doi:10.1186/s12985-023-02195-9)
Supplement: Supplementary file 1 — Additional file 1: Supplementary Table S1. Data imported from BPS for each patient: code and definition of the variable. Supplementary Table S2. Log Hazard ratios obtained for the drugs tested, along with standard deviations (SDs), upper and lower coefficient intervals (CI), nominal and FDR-adjusted p-values. Also, Lymphocyte proliferation values (see Methods) along with standard deviations (SDs), upper and lower coefficient intervals (CI), nominal and FDR-adjusted p-values. The two last columns indicate the drugs used in the machine learning drug repurposing prediction study and the proteins targeted by the drug. Targets marked with an * were those analyzed in the machine learning drug repurposing [file 12985_2023_2195_MOESM1_ESM.pdf]

Real-world evidence with a retrospective cohort of 15,968 COVID-19 hospitalized patients suggests 21 new effective treatments.

Carlos Loucera, Rosario Carmona, Marina Esteban-Medina, Gerrit Bostelmann, Dolores Muñoyerro-Muñiz, Román Villegas, María Peña-Chilet, Joaquin Dopazo

Supplementary Material

Contents

Table S1 2

Table S2 3

Table S1. Data imported from BPS for each patient: code and definition of the variable.

Code	Meaning
FECNAC	Birth date
FECDEF	Death date
SEXO	Gender
FEC_INGRESO	Hospital admission date
FEC_ALTA	Discharge date
MOTIVO_ALTA	Reason for the discharge: (recovery/death/admission in another hospital/voluntary discharge/retirement home/unspecified)
COD_PATOLOGIA_CRONICA	Hospital codes for chronic conditions
COD_FEC_INI_PATOLOGIA	Date of condition diagnosis
COD_CIE_NORMALIZADO	A mixture of ICD9 and ICD10 codes for diseases
DESC_CIE_NORMALIZADO	Description of the ICD
FECINI_DIAG	Diagnosis date
FECFIN_DIAG	End of the diagnosed condition
FUENTE_DIAG	Source of the diagnosis (hospital, emergency, etc.)
IND_CRONICO_HCUP	Is a chronic disease? (yes/no)
Test COVID: FECHA	Test COVID date
Test COVID: TYPE	PCR / antigens
Test COVID: RESULTADO_TEST	Result of the test (positive/negative)
Pharmacy (Hospital and external): DESCRIPCION	List of drugs used in hospital or purchased in the pharmacies
Pharmacy (Hospital and external): FECHA	Dispensing date
VACUNA	List of vaccines
VACUNAFECHA	Vaccination dates
COD_CLC	List of analytical tests
FEC_EXTRAE	Analytic test date
VALOR	Analytic test value

Table S2. Log Hazard ratios obtained for the drugs tested, along with standard deviations (SDs), upper and lower coefficient intervals (CI), nominal and FDR-adjusted p-values. Also, Lymphocyte proliferation values (see Methods) along with standard deviations (SDs), upper and lower coefficient intervals (CI), nominal and FDR-adjusted p-values. The two last columns indicate the drugs used in the machine learning drug repurposing prediction study and the proteins targeted by the drug. Targets marked with an * were those analyzed in the machine learning drug repurposing.

Drugbank ID	name	N	LHR estimate	LHR SD	LHR CI 5%	LHR CI 95%	LHR p-value	LHR FDR p-value	Lymphocyte counr	LC CI 5%	LC CI 95%	LC p-value	LC FDR p-value	ML prediction	Targets
**DB00047**	Insulin glargine	565	-0.15163	0.10438	-0.35620	0.05295	0.14631	0.27890	1.10043	-0.00230	0.00816	0.27162	0.41946	No	IGF1R, INSR
**DB00115**	Cyanocobalamin	400	-0.00558	0.14123	-0.28238	0.27122	0.96850	0.97596	0.07283	-0.00843	0.00903	0.94198	0.94198	No	MMAA, MMACHC, MMUT, MTHFR, MTR, MTRR
**DB00126**	Ascorbic acid	840	-0.33321	0.10674	-0.54241	-0.12400	0.00180	0.01219	3.66682	0.00406	0.01344	0.00026	0.00127	Yes	P3H1^{+}, P4HTM^{+}, PHYH^{+}, TMLHE^{+}, ALKBH2, ALKBH3, BBOX1, DBH, EGLN1, EGLN2, EGLN3, KDM5D, OGFOD1, OGFOD2, P3H2, P3H3, P4HA1, PAM, PLOD1, PLOD2, PLOD3
**DB00128**	Aspartic acid	648	-0.29845	0.13140	-0.55598	-0.04091	0.02313	0.06911	3.66511	0.00543	0.01791	0.00027	0.00127	Yes	ASNS^{+}, ACY1, ADSS1, ADSS2, ASPA, ASPH, ASRGL1, ASS1, DARS1, DARS2, GOT1, GOT2, LYZ, PAICS, RNASE1, SLC1A1, SLC25A12, SLC25A13
**DB00146**	Calcifediol	530	-0.40717	0.15363	-0.70827	-0.10607	0.00804	0.03164	3.40110	0.00502	0.01869	0.00073	0.00328	Yes	VDR^{+}
**DB00158**	Folic acid	624	-0.01488	0.10223	-0.21525	0.18549	0.88431	0.95713	-0.66950	-0.00805	0.00395	0.50345	0.63980	Yes	FOLR1^{+}, FOLR2, FOLR3
**DB00169**	Cholecalciferol	725	-0.28067	0.10502	-0.48651	-0.07483	0.00753	0.03062	3.66953	0.00437	0.01440	0.00026	0.00127	Yes	VDR^{+}
**DB00177**	Valsartan	383	-0.12361	0.12964	-0.37769	0.13047	0.34033	0.51259	1.85808	-0.00034	0.01259	0.06391	0.14444	Yes	AGTR1^{+}
**DB00178**	Ramipril	509	-0.26557	0.11700	-0.49489	-0.03624	0.02323	0.06911	2.29420	0.00102	0.01294	0.02222	0.06025	No	ACE, BDKRB1
**DB00186**	Lorazepam	1057	-0.22996	0.08503	-0.39662	-0.06330	0.00684	0.02879	1.16166	-0.00180	0.00705	0.24568	0.39964	No	GABRA1, GABRA2, GABRA3, GABRA4, GABRA5, GABRA6, GABRB1, GABRB2, GABRB3, GABRD, GABRE, GABRG1, GABRG2, GABRG3, GABRP, GABRQ

Drugbank ID	name	N	LHR estimate	LHR SD	LHR CI 5%	LHR CI 95%	LHR p-value	LHR FDR p-value	Lymphocyte counr	LC CI 5%	LC CI 95%	LC p-value	LC FDR p-value	ML prediction	Targets
**DB00193**	Tramadol	949	-0.16897	0.08313	-0.33191	-0.00604	0.04209	0.11921	3.16350	0.00252	0.01072	0.00162	0.00636	Yes	SLC6A4⁺, ADORA1, ADRA2A, ADRA2B, ADRA2C, CHRM1, CHRM3, GRIN1, GRIN2A, GRIN2B, GRIN2C, GRIN2D, GRIN3A, GRIN3B, HTR2C, OPRD1, OPRK1, OPRM1, SCN2A, SLC6A2, TACR1, TRPV1
**DB00207**	Azithromycin	2465	-0.16216	0.05779	-0.27542	-0.04889	0.00502	0.02267	4.36750	0.00338	0.00888	0.00001	0.00018	No	PADI4
**DB00213**	Pantoprazole	476	0.05155	0.10359	-0.15149	0.25459	0.61874	0.78589	1.70433	-0.00079	0.01131	0.08903	0.18727	No	ATP4A, DDAH1
**DB00214**	Torasemide	278	0.07079	0.13233	-0.18858	0.33016	0.59267	0.77748	1.21943	-0.00296	0.01270	0.22380	0.37346	No	SLC12A1, SLC12A2
**DB00215**	Citalopram	300	-0.13647	0.14275	-0.41625	0.14331	0.33907	0.51259	2.85283	0.00363	0.01967	0.00464	0.01666	Yes	HRH1⁺, SLC6A4⁺
**DB00230**	Pregabalin	382	0.04019	0.11379	-0.18284	0.26321	0.72397	0.85752	-0.37733	-0.00766	0.00518	0.70612	0.76659	No	CACNA2D1
**DB00275**	Olmesartan	413	-0.36562	0.12879	-0.61803	-0.11320	0.00453	0.02209	2.37903	0.00137	0.01416	0.01786	0.05002	Yes	AGTR1⁺
**DB00316**	Acetaminophen	7258	-0.27573	0.04035	-0.35481	-0.19665	0.00000	0.00000	6.53570	0.00361	0.00671	0.00000	0.00000	Yes	PTGS2⁺, PTGES3, PTGS1, TRPV1
**DB00318**	Codeine	1019	-0.29747	0.09647	-0.48655	-0.10840	0.00204	0.01247	3.92417	0.00441	0.01324	0.00009	0.00073	No	OPRD1, OPRK1, OPRM1
**DB00321**	Amitriptyline	162	0.00881	0.18668	-0.35708	0.37471	0.96235	0.97596	1.72008	-0.00132	0.01939	0.08746	0.18719	Yes	HRH1⁺, HTR1B⁺, NTRK1⁺, SLC6A4⁺, ADRA1A, ADRA1B, ADRA1D, ADRA2A, CHRM1, CHRM2, CHRM3, CHRM4, CHRM5, HRH2, HRH4, HTR1A, HTR1D, HTR2A, HTR2C, HTR6, HTR7, KCNA1, KCNH2, KCNH6, KCNH7, KCNQ2, KCNQ3, NTRK2, OPRD1, OPRK1, OPRM1, SIGMAR1, SLC6A2
**DB00331**	Metformin	1896	-0.35648	0.06733	-0.48845	-0.22451	0.00000	0.00000	3.88744	0.00273	0.00827	0.00010	0.00073	Yes	GPD1⁺, ETFDH, PRKAB1
**DB00332**	Ipratropium	1322	-0.04102	0.07479	-0.18760	0.10557	0.58340	0.77364	5.03850	0.00632	0.01436	0.00000	0.00001	No	CHRM1, CHRM2, CHRM3
**DB00335**	Atenolol	187	-0.31536	0.19350	-0.69460	0.06389	0.10315	0.21696	-0.60431	-0.01264	0.00669	0.54638	0.66658	No	ADRB1, ADRB2
**DB00338**	Omeprazole	5173	-0.20373	0.04349	-0.28897	-0.11848	0.00000	0.00004	3.92296	0.00189	0.00566	0.00009	0.00073	No	AHR, ATP4A
**DB00341**	Cetirizine	233	-0.70557	0.22786	-1.15217	-0.25896	0.00196	0.01247	0.99063	-0.00462	0.01405	0.32232	0.45199	Yes	HRH1⁺

Drugbank ID	name	N	LHR estimate	LHR SD	LHR CI 5%	LHR CI 95%	LHR p-value	LHR FDR p-value	Lymphocyte counr	LC CI 5%	LC CI 95%	LC p-value	LC FDR p-value	ML prediction	Targets
**DB00370**	Mirtazapine	201	0.05217	0.15216	-0.24605	0.35040	0.73168	0.85831	-0.38123	-0.01043	0.00705	0.70345	0.76659	Yes	HRH1^{+}, ADRA1A, ADRA1B, ADRA1D, ADRA2A, HTR2A, HTR2C, OPRK1
**DB00373**	Timolol	254	-0.21656	0.14751	-0.50567	0.07255	0.14207	0.27512	-0.37196	-0.01184	0.00806	0.71004	0.76659	No	ADRB1, ADRB2
**DB00381**	Amlodipine	1250	-0.27091	0.07289	-0.41378	-0.12804	0.00020	0.00190	1.84840	-0.00020	0.00666	0.06479	0.14444	Yes	CACNA1B^{+}, CA1, CACNA1C, CACNA1I, CACNA2D3, CACNB1, SMPD1
**DB00394**	Beclomethasone dipropionate	792	-0.36901	0.11319	-0.59085	-0.14717	0.00111	0.00849	4.15390	0.00594	0.01656	0.00004	0.00045	No	NR3C1
**DB00404**	Alprazolam	557	-0.00579	0.10444	-0.21049	0.19891	0.95578	0.97596	3.28606	0.00368	0.01455	0.00109	0.00460	Yes	GABRA2^{+}, GABRA5^{+}, GABRA6^{+}, GABRA1, GABRA3, GABRA4, GABRB1, GABRB2, GABRB3, GABRD, GABRE, GABRG1, GABRG2, GABRG3, GABRP, GABRQ
**DB00421**	Spironolactone	272	0.12328	0.12368	-0.11914	0.36569	0.31891	0.50226	0.44874	-0.00510	0.00815	0.65401	0.75990	Yes	CACNG1^{+}, NR1I2^{+}, AR, CACNA1A, CACNA1B, CACNA1C, CACNA1D, CACNA1E, CACNA1F, CACNA1G, CACNA1H, CACNA1I, CACNA1S, CACNA2D1, CACNA2D2, CACNA2D3, CACNA2D4, CACNB1, CACNB2, CACNB3, CACNB4, CACNG2, CACNG3, CACNG4, CACNG5, CACNG6, CACNG7, CACNG8, CYP11B2, CYP17A1, NR3C1, NR3C2, PGR, SHBG, SRD5A1, SRD5A2, SRD5A3
**DB00425**	Zolpidem	292	-0.00952	0.13596	-0.27599	0.25694	0.94415	0.97596	1.81929	-0.00058	0.01460	0.07037	0.15332	Yes	GABRA2^{+}, GABRA1, GABRA3, GABRG2
**DB00437**	Allopurinol	511	-0.06858	0.10105	-0.26662	0.12947	0.49736	0.70555	-0.61971	-0.00671	0.00349	0.53579	0.66026	No	XDH
**DB00440**	Trimethoprim	161	-0.10459	0.21128	-0.51870	0.30951	0.62057	0.78589	2.43774	0.00277	0.02537	0.01575	0.04803	No	
**DB00448**	Lansoprazole	236	-0.18294	0.15103	-0.47895	0.11306	0.22577	0.38255	-0.32776	-0.01012	0.00721	0.74337	0.78862	No	ATP4A, MAPT

Drugbank ID	name	N	LHR estimate	LHR SD	LHR CI 5%	LHR CI 95%	LHR p-value	LHR FDR p-value	Lymphocyte counr	LC CI 5%	LC CI 95%	LC p-value	LC FDR p-value	ML prediction	Targets
**DB00451**	Levothyroxine	396	-0.22450	0.15086	-0.52017	0.07118	0.13671	0.26902	2.26495	0.00109	0.01517	0.02414	0.06177	Yes	THRB^{+}, ITGAV, ITGB3, THRA
**DB00455**	Loratadine	251	-0.36187	0.18521	-0.72487	0.00112	0.05071	0.12890	1.33779	-0.00295	0.01552	0.18172	0.33090	Yes	HRH1^{+}, KCNH2
**DB00502**	Haloperidol	206	0.03799	0.14360	-0.24346	0.31945	0.79134	0.91130	1.85439	-0.00042	0.01528	0.06511	0.14444	No	ADRA1A, ADRA2A, ADRA2B, ADRA2C, CHRM3, DRD1, DRD2, DRD3, GRIN2B, HRH1, HTR1A, HTR2A, HTR2C, HTR6, HTR7, MCHR1, SIGMAR1, SLC18A2
**DB00503**	Ritonavir	199	-2.56715	1.01835	-4.56309	-0.57122	0.01171	0.04328	0.54421	-0.00734	0.01298	0.58700	0.69528	Yes	NR1I2^{+}
**DB00537**	Ciprofloxacin	353	0.22127	0.11505	-0.00423	0.44676	0.05445	0.13286	1.05222	-0.00353	0.01174	0.29339	0.44189	No	KCNH2, TOP2A
**DB00584**	Enalapril	1598	-0.19957	0.06725	-0.33138	-0.06775	0.00300	0.01745	3.76049	0.00297	0.00945	0.00018	0.00103	No	ACE
**DB00590**	Doxazosin	428	-0.14507	0.11283	-0.36622	0.07607	0.19853	0.35103	1.09164	-0.00271	0.00953	0.27555	0.42021	No	ADRA1A, ADRA1B, ADRA1D, KCNH2, KCNH6, KCNH7
**DB00612**	Bisoprolol	1409	0.03952	0.06188	-0.08175	0.16080	0.52296	0.72502	0.45436	-0.00261	0.00419	0.64965	0.75990	No	ADRB1, ADRB2
**DB00628**	Clorazepic acid	330	-0.03228	0.12859	-0.28432	0.21976	0.80180	0.91421	0.63151	-0.00477	0.00934	0.52815	0.66026	No	GABRA1, GABRA2, GABRA3, GABRA4, GABRA5, GABRA6, GABRB1, GABRB2, GABRB3, GABRD, GABRE, GABRG1, GABRG2, GABRG3, GABRP, GABRQ
**DB00630**	Alendronic acid	172	-0.08885	0.18170	-0.44496	0.26727	0.62485	0.78589	1.29554	-0.00392	0.01921	0.19684	0.35316	No	ATP6V1A, FDPS, PTPN4, PTPRE, PTPRS
**DB00635**	Prednisone	1818	-0.35982	0.08332	-0.52311	-0.19652	0.00002	0.00018	5.43363	0.00644	0.01371	0.00000	0.00000	No	NR3C1
**DB00641**	Simvastatin	1588	-0.40558	0.07026	-0.54328	-0.26789	0.00000	0.00000	5.16632	0.00523	0.01163	0.00000	0.00001	Yes	ITGAL^{+}, HDAC2, HMGCR
**DB00646**	Nystatin	165	-0.12519	0.20590	-0.52875	0.27836	0.54317	0.73629	-1.11099	-0.01934	0.00540	0.26787	0.41897	No	
**DB00654**	Latanoprost	183	0.28492	0.14750	-0.00418	0.57402	0.05341	0.13286	-0.93373	-0.01427	0.00483	0.35214	0.48271	No	PTGFR
**DB00656**	Trazodone	468	0.17836	0.09654	-0.01086	0.36758	0.06468	0.14985	0.26362	-0.00534	0.00701	0.79221	0.81906	Yes	HRH1^{+}, SLC6A4^{+}, ADRA1A, ADRA2A, HTR1A, HTR2A, HTR2C
**DB00678**	Losartan	1409	-0.10898	0.06648	-0.23928	0.02132	0.10114	0.21648	3.90438	0.00328	0.00991	0.00010	0.00073	Yes	AGTR1^{+}
**DB00695**	Furosemide	1920	0.24131	0.05212	0.13916	0.34346	0.00000	0.00005	-3.74328	-0.00805	-0.00251	0.00019	0.00103	No	CA2, GPR35, SLC12A1

Drugbank ID	name	N	LHR estimate	LHR SD	LHR CI 5%	LHR CI 95%	LHR p-value	LHR FDR p-value	Lymphocyte counr	LC CI 5%	LC CI 95%	LC p-value	LC FDR p-value	ML prediction	Targets
**DB00706**	Tamsulosin	1080	0.19252	0.06766	0.05991	0.32513	0.00444	0.02209	0.96661	-0.00185	0.00546	0.33397	0.46300	No	ADRA1A, ADRA1B, ADRA1D
**DB00715**	Paroxetine	174	-0.02896	0.19600	-0.41311	0.35518	0.88252	0.95713	1.21415	-0.00374	0.01596	0.22631	0.37346	Yes	SLC6A4^{+}, ADRA1A, ADRA1B, ADRA1D, ADRA2A, ADRA2B, ADRA2C, ADRB1, ADRB2, ADRB3, CHRM1, CHRM2, CHRM3, CHRM4, CHRM5, DRD1, DRD2, DRD5, HRH1, HTR1A, HTR1B, HTR1D, HTR1E, HTR1F, HTR2A, HTR2B, HTR2C, HTR3A, HTR3B, HTR3C, HTR3D, HTR3E, HTR4, HTR6, HTR7, SLC6A2
**DB00727**	Nitroglycerin	359	0.20588	0.11364	-0.01684	0.42860	0.07002	0.15532	-1.28181	-0.01149	0.00239	0.20108	0.35553	No	NPR1
**DB00736**	Esomeprazole	277	-0.26589	0.15735	-0.57428	0.04251	0.09106	0.19839	1.55751	-0.00180	0.01576	0.12041	0.24082	No	ATP4A, DDAH1
**DB00758**	Clopidogrel	285	0.05387	0.12828	-0.19754	0.30529	0.67450	0.81551	-0.39382	-0.00830	0.00552	0.69405	0.76659	No	P2RY12
**DB00766**	Clavulanic acid	779	0.03666	0.09079	-0.14128	0.21461	0.68635	0.82093	1.03869	-0.00255	0.00831	0.29945	0.44230	No	
**DB00788**	Naproxen	223	-0.29150	0.21007	-0.70324	0.12023	0.16525	0.30547	3.21950	0.00592	0.02428	0.00154	0.00625	Yes	PTGS2^{+}, PTGS1
**DB00796**	Candesartan cilexetil	198	-0.30312	0.19515	-0.68561	0.07938	0.12037	0.24890	2.51961	0.00308	0.02457	0.01261	0.04049	Yes	AGTR1^{+}
**DB00806**	Pentoxifylline	231	-0.01548	0.13760	-0.28517	0.25420	0.91040	0.96582	-0.21323	-0.00883	0.00709	0.83135	0.85231	Yes	NT5E^{+}, ADORA1, ADORA2A
**DB00813**	Fentanyl	251	0.16883	0.12628	-0.07868	0.41633	0.18124	0.32517	0.12960	-0.00829	0.00949	0.89698	0.91193	No	ABCB1, OPRD1, OPRK1, OPRM1
**DB00828**	Fosfomycin	298	-0.12532	0.14617	-0.41181	0.16118	0.39127	0.57512	0.54516	-0.00530	0.00942	0.58622	0.69528	No	
**DB00829**	Diazepam	641	-0.41669	0.12112	-0.65408	-0.17930	0.00058	0.00473	2.16427	0.00057	0.01141	0.03084	0.07525	Yes	GABRA2^{+}, GABRA5^{+}, GABRD^{+}, GABRA1, GABRA3, GABRA4, GABRA6, GABRB1, GABRB2, GABRB3, GABRE, GABRG1, GABRG2, GABRG3, GABRP, GABRQ
**DB00927**	Famotidine	207	-0.19262	0.16794	-0.52177	0.13653	0.25139	0.40892	0.57316	-0.00725	0.01320	0.56698	0.68487	No	HRH2
**DB00938**	Salmeterol	279	0.00481	0.13478	-0.25935	0.26897	0.97154	0.97596	1.15088	-0.00355	0.01353	0.25073	0.40250	No	ADRB1, ADRB2, ADRB3
**DB00945**	Acetylsalicylic acid	2127	-0.19543	0.05548	-0.30416	-0.08670	0.00043	0.00372	1.40216	-0.00080	0.00482	0.16103	0.30695	Yes	CASP1^{+}, CCND1^{+}, IKBKB^{+}, MYC^{+}, NFKBIA^{+}, PTGS2^{+},

Drugbank ID	name	N	LHR estimate	LHR SD	LHR CI 5%	LHR CI 95%	LHR p-value	LHR FDR p-value	Lymphocyte counr	LC CI 5%	LC CI 95%	LC p-value	LC FDR p-value	ML prediction	Targets
															RPS6KA3^{+}, AKR1C1, CASP3, EDNRA, HSPA5, MAPK1, MAPK15, MAPK3, MAPK4, MAPK6, MAPK7, PCNA, PRKAA1, PRKAA2, PRKAB1, PRKAB2, PRKAG1, PRKAG2, PRKAG3, PTGS1, TNFAIP6, TP53
**DB00966**	Telmisartan	226	-0.09613	0.15655	-0.40296	0.21070	0.53917	0.73629	1.25784	-0.00308	0.01409	0.20987	0.36520	Yes	AGTR1^{+}, PPARG^{+}
**DB00973**	Ezetimibe	344	-0.16155	0.13076	-0.41783	0.09473	0.21665	0.37227	1.86701	-0.00040	0.01628	0.06262	0.14444	Yes	SOAT1^{+}, ANPEP, NPC1L1
**DB00983**	Formoterol	1380	-0.37040	0.08596	-0.53888	-0.20192	0.00002	0.00018	5.13579	0.00650	0.01453	0.00000	0.00001	No	ADRB1, ADRB2, ADRB3
**DB00986**	Glycopyrronium	196	0.20218	0.14234	-0.07681	0.48116	0.15550	0.29187	0.40028	-0.00780	0.01181	0.68940	0.76659	No	CHRM1, CHRM2, CHRM3
**DB00999**	Hydrochlorothiazide	2197	-0.35148	0.06036	-0.46979	-0.23318	0.00000	0.00000	3.98046	0.00273	0.00802	0.00007	0.00067	Yes	KCNMA1^{+}, SLC12A3
**DB01001**	Salbutamol	670	-0.11964	0.10967	-0.33459	0.09530	0.27528	0.44190	3.90623	0.00567	0.01710	0.00011	0.00073	No	ADRB1, ADRB2, ADRB3
**DB01029**	Irbesartan	184	-0.49710	0.21193	-0.91248	-0.08171	0.01900	0.06623	0.68108	-0.00656	0.01359	0.49622	0.63725	Yes	AGTR1^{+}, JUN^{+}
**DB01039**	Fenofibrate	244	-0.53853	0.20520	-0.94072	-0.13635	0.00868	0.03309	1.49896	-0.00196	0.01472	0.13512	0.26589	Yes	NR1I2^{+}, MMP25, PPARA
**DB01050**	Ibuprofen	519	-0.65959	0.20808	-1.06743	-0.25175	0.00153	0.01095	2.18280	0.00080	0.01503	0.02961	0.07372	Yes	BCL2^{+}, CFTR^{+}, PPARG^{+}, PTGS2^{+}, FABP2, GP1BA, PPARA, PTGS1, S100A7, THBD
**DB01060**	Amoxicillin	1090	0.00515	0.07787	-0.14746	0.15777	0.94725	0.97596	1.24767	-0.00151	0.00681	0.21253	0.36520	No	
**DB01068**	Clonazepam	167	0.07509	0.17917	-0.27608	0.42627	0.67513	0.81551	-0.11898	-0.01232	0.01095	0.90540	0.91288	Yes	NR1I2^{+}, GABRA1, GABRA2, GABRA3, GABRA4, GABRA5, GABRA6, GABRB1, GABRB2, GABRB3, GABRD, GABRE, GABRG1, GABRG2, GABRG3, GABRP, GABRQ
**DB01076**	Atorvastatin	1112	-0.08449	0.07355	-0.22864	0.05966	0.25066	0.40892	1.14066	-0.00164	0.00621	0.25429	0.40289	Yes	DPP4^{+}, AHR, HDAC2, HMGCR, NR1I3
**DB01098**	Rosuvastatin	314	-0.30099	0.14942	-0.59386	-0.00813	0.04397	0.11921	1.02993	-0.00378	0.01210	0.30385	0.44230	No	HMGCR, ITGAL
**DB01104**	Sertraline	359	-0.00366	0.12135	-0.24149	0.23418	0.97596	0.97596	2.98406	0.00362	0.01747	0.00303	0.01156	Yes	SLC6A4^{+}, PGRMC1, SIGMAR1, SLC29A4, SLC6A2, SLC6A3
**DB01112**	Cefuroxime	352	0.06282	0.13187	-0.19565	0.32129	0.63381	0.78903	1.21146	-0.00280	0.01190	0.22652	0.37346	No	

Drugbank ID	name	N	LHR estimate	LHR SD	LHR CI 5%	LHR CI 95%	LHR p-value	LHR FDR p-value	Lymphocyte counr	LC CI 5%	LC CI 95%	LC p-value	LC FDR p-value	ML prediction	Targets
**DB01120**	Gliclazide	322	-0.41723	0.15098	-0.71315	-0.12131	0.00572	0.02492	2.74039	0.00257	0.01549	0.00649	0.02262	No	ABCC8, VEGFA
**DB01126**	Dutasteride	547	0.16492	0.08814	-0.00782	0.33766	0.06132	0.14668	1.42431	-0.00139	0.00878	0.15492	0.30001	Yes	SRD5A1^{+}, SRD5A2
**DB01136**	Carvedilol	370	-0.03093	0.11715	-0.26054	0.19868	0.79178	0.91130	-0.99171	-0.00925	0.00303	0.32204	0.45199	Yes	HIF1A^{+}, ADRA1A, ADRA1B, ADRA1D, ADRA2A, ADRA2B, ADRA2C, ADRB1, ADRB2, GJA1, KCNH2, KCNJ2, KCNJ4, NDUFC2, NPPB, SELE, VCAM1, VEGFA
**DB01137**	Levofloxacin	1052	0.03795	0.07490	-0.10885	0.18475	0.61239	0.78589	3.76225	0.00391	0.01243	0.00018	0.00103	No	
**DB01175**	Escitalopram	175	0.20050	0.16727	-0.12734	0.52834	0.23066	0.38549	2.89171	0.00553	0.02885	0.00436	0.01613	Yes	HRH1^{+}, SLC6A4^{+}, ADRA1A, ADRA1B, ADRA1D, ADRA2A, ADRA2B, ADRA2C, CHRM1, DRD2, HTR1A, HTR2A, HTR2C, SLC6A2, SLC6A3
**DB01184**	Domperidone	226	0.13252	0.14828	-0.15811	0.42315	0.37149	0.55270	1.64601	-0.00154	0.01740	0.10098	0.20881	No	DRD2, DRD3
**DB01222**	Budesonide	1047	-0.25260	0.08800	-0.42508	-0.08012	0.00410	0.02174	3.28003	0.00289	0.01151	0.00108	0.00460	No	ANXA1, NR3C1
**DB01225**	Enoxaparin	3237	-1.20921	0.10316	-1.41141	-1.00702	0.00000	0.00000	11.31073	0.01233	0.01750	0.00000	0.00000	No	F10, SERPINC1
**DB01233**	Metoclopramide	498	0.05646	0.12341	-0.18542	0.29835	0.64729	0.79767	2.38020	0.00145	0.01496	0.01767	0.05002	Yes	HTR4^{+}, CHRM1, DRD2, HTR3A
**DB01234**	Dexamethasone	2469	-0.75679	0.10273	-0.95813	-0.55545	0.00000	0.00000	9.42789	0.01233	0.01881	0.00000	0.00000	Yes	NR1I2^{+}, ANXA1, NOS2, NR0B1, NR3C1
**DB01261**	Sitagliptin	448	-0.36512	0.12515	-0.61040	-0.11984	0.00353	0.01956	2.56016	0.00179	0.01355	0.01077	0.03552	Yes	DPP4^{+}
**DB01306**	Insulin aspart	214	-0.33848	0.18350	-0.69814	0.02117	0.06510	0.14985	2.04618	0.00041	0.01859	0.04196	0.10038	No	IGF1R, INSR
**DB01373**	Calcium	641	-0.30728	0.10895	-0.52082	-0.09375	0.00480	0.02250	3.75161	0.00479	0.01529	0.00019	0.00103	Yes	ALPP^{+}, CAST^{+}, COMP^{+}, PCDH19^{+}, PDCD6^{+}, S100A8^{+}, S100B^{+}, AOC1, ATP2C1, BMP4, CACNA1C, CALM3, CP, MGP, S100A13, S100A2, S100A9, SPTBN1, TNNC1, TNNC2
**DB01409**	Tiotropium	449	-0.01915	0.10911	-0.23299	0.19470	0.86068	0.95458	0.43126	-0.00476	0.00746	0.66649	0.76088	No	CHRM1, CHRM2, CHRM3, CHRM4, CHRM5
**DB01418**	Acenocoumarol	511	0.17844	0.08974	0.00254	0.35433	0.04678	0.12407	-0.74581	-0.00788	0.00353	0.45615	0.59670	No	VKORC1

Drugbank ID	name	N	LHR estimate	LHR SD	LHR CI 5%	LHR CI 95%	LHR p-value	LHR FDR p-value	Lymphocyte counr	LC CI 5%	LC CI 95%	LC p-value	LC FDR p-value	ML prediction	Targets
**DB01558**	Bromazepam	471	-0.11470	0.11560	-0.34127	0.11188	0.32112	0.50226	2.71790	0.00234	0.01446	0.00682	0.02312	Yes	GABRA2^{+}, GABRA5^{+}, GABRA6^{+}, GABRD^{+}, GABRA1, GABRA3, GABRA4, GABRB1, GABRB2, GABRB3, GABRE, GABRG1, GABRG2, GABRG3, GABRP, GABRQ
**DB01592**	Iron	959	0.07574	0.07736	-0.07588	0.22736	0.32752	0.50579	-1.34037	-0.00790	0.00148	0.18047	0.33090	Yes	FEN1^{+}, AHSP, CP, EGLN1, FTH1, FXN, HBA1, HDAC8, NEIL1, NEIL2, POLB, TF, TFRC
**DB01601**	Lopinavir	185	-2.47282	1.01507	-4.46233	-0.48332	0.01485	0.05327	1.00651	-0.00511	0.01591	0.31573	0.45199	No	
**DB01611**	Hydroxychloroquine	320	-1.17992	0.31635	-1.79995	-0.55988	0.00019	0.00190	-0.28913	-0.00963	0.00729	0.77293	0.81291	Yes	TLR7^{+}, ACE2, TLR9
**DB04817**	Metamizole	2843	-0.06400	0.05075	-0.16347	0.03546	0.20723	0.36117	4.06537	0.00267	0.00763	0.00005	0.00055	No	PTGS1
**DB04876**	Vildagliptin	200	-0.13189	0.16107	-0.44757	0.18379	0.41287	0.59965	2.38357	0.00176	0.01809	0.01804	0.05002	Yes	DPP4^{+}
**DB06228**	Rivaroxaban	200	-0.09436	0.16292	-0.41368	0.22497	0.56249	0.75411	0.77397	-0.00583	0.01340	0.43986	0.58970	No	F10
**DB06292**	Dapagliflozin	229	-0.35373	0.19329	-0.73256	0.02510	0.06724	0.15191	2.50511	0.00236	0.01940	0.01312	0.04105	No	SLC5A2
**DB06605**	Apixaban	345	-0.01609	0.11841	-0.24817	0.21599	0.89191	0.95713	0.74036	-0.00432	0.00955	0.45975	0.59670	No	F10
**DB06698**	Betahistine	409	-0.01532	0.11542	-0.24154	0.21089	0.89437	0.95713	1.38293	-0.00188	0.01093	0.16755	0.31447	Yes	HRH1^{+}, HRH3
**DB08810**	Cinitapride	210	-0.04103	0.17829	-0.39046	0.30840	0.81799	0.92402	2.41071	0.00217	0.02098	0.01681	0.05002	No	HTR1A, HTR2A, HTR4
**DB08882**	Linagliptin	306	0.17434	0.11700	-0.05498	0.40366	0.13620	0.26902	-0.43021	-0.00919	0.00588	0.66733	0.76088	Yes	DPP4^{+}
**DB08906**	Fluticasone furoate	662	-0.20888	0.10361	-0.41196	-0.00580	0.04381	0.11921	4.00962	0.00547	0.01595	0.00007	0.00067	No	NR3C1, NR3C2, PGR
**DB09038**	Empagliflozin	239	-0.42376	0.18355	-0.78352	-0.06401	0.02096	0.06730	2.26829	0.00126	0.01714	0.02430	0.06177	No	SLC5A2
**DB09082**	Vilanterol	328	-0.20516	0.14917	-0.49754	0.08722	0.16904	0.30780	2.28033	0.00124	0.01648	0.02336	0.06177	No	ADRB2
**DB09153**	Sodium chloride	381	-0.29602	0.13845	-0.56739	-0.02466	0.03251	0.09443	0.62571	-0.00479	0.00929	0.53189	0.66026	No	
**DB09154**	Sodium citrate	272	-0.41841	0.18019	-0.77158	-0.06523	0.02023	0.06703	0.88022	-0.00453	0.01193	0.37952	0.51446	No	
**DB09214**	Dexketoprofen	327	-0.25471	0.17102	-0.58990	0.08048	0.13639	0.26902	1.02841	-0.00427	0.01369	0.30453	0.44230	Yes	PTGS2^{+}, PTGS1
**DB09238**	Manidipine	160	-0.13985	0.18858	-0.50945	0.22975	0.45832	0.65782	-0.34343	-0.01189	0.00835	0.73174	0.78309	No	CACNA1A, CACNA1C, CACNA1D, CACNA1F, CACNA1G, CACNA1H, CACNA1I, CACNA1S, CACNB1, CACNB2, CACNB3, CACNB4

Drugbank ID	name	N	LHR estimate	LHR SD	LHR CI 5%	LHR CI 95%	LHR p-value	LHR FDR p-value	Lymphocyte counr	LC CI 5%	LC CI 95%	LC p-value	LC FDR p-value	ML prediction	Targets
**DB09341**	Dextrose, unspecified form	311	-0.37999	0.16377	-0.70099	-0.05900	0.02033	0.06703	0.75441	-0.00458	0.01031	0.45117	0.59670	No	
**DB11921**	Deflazacort	629	-0.06549	0.10221	-0.26581	0.13482	0.52164	0.72502	3.82838	0.00522	0.01618	0.00014	0.00092	No	NR3C1
**DB13872**	Lormetazepam	806	0.15230	0.07786	-0.00029	0.30490	0.05044	0.12890	0.37198	-0.00367	0.00540	0.71001	0.76659	Yes	GABRA2^{+}, GABRA5^{+}, GABRA1, GABRA3, GABRA4, GABRA6, GABRB1, GABRB2, GABRB3, GABRD, GABRE, GABRG1, GABRG2, GABRG3, GABRP, GABRQ
**DB14500**	Potassium	352	-0.34762	0.15244	-0.64641	-0.04884	0.02259	0.06911	0.27568	-0.00602	0.00798	0.78296	0.81642	No	ATP1A1
**DB14761**	Remdesivir	190	-0.03958	0.20608	-0.44348	0.36432	0.84768	0.94878	1.57875	-0.00178	0.01610	0.11603	0.23592	No	
